# Supplementary material for: A mitotic bookmark coordinates transcription and replication in Drosophila embryos
Source: Nucleic Acids Res. 2025 Dec 31;53(22):gkaf1429. doi: 10.1093/nar/gkaf1429 (PMC12754778; doi:10.1093/nar/gkaf1429)
Supplement: gkaf1429_Supplemental_File [file gkaf1429_supplemental_file.docx]

**
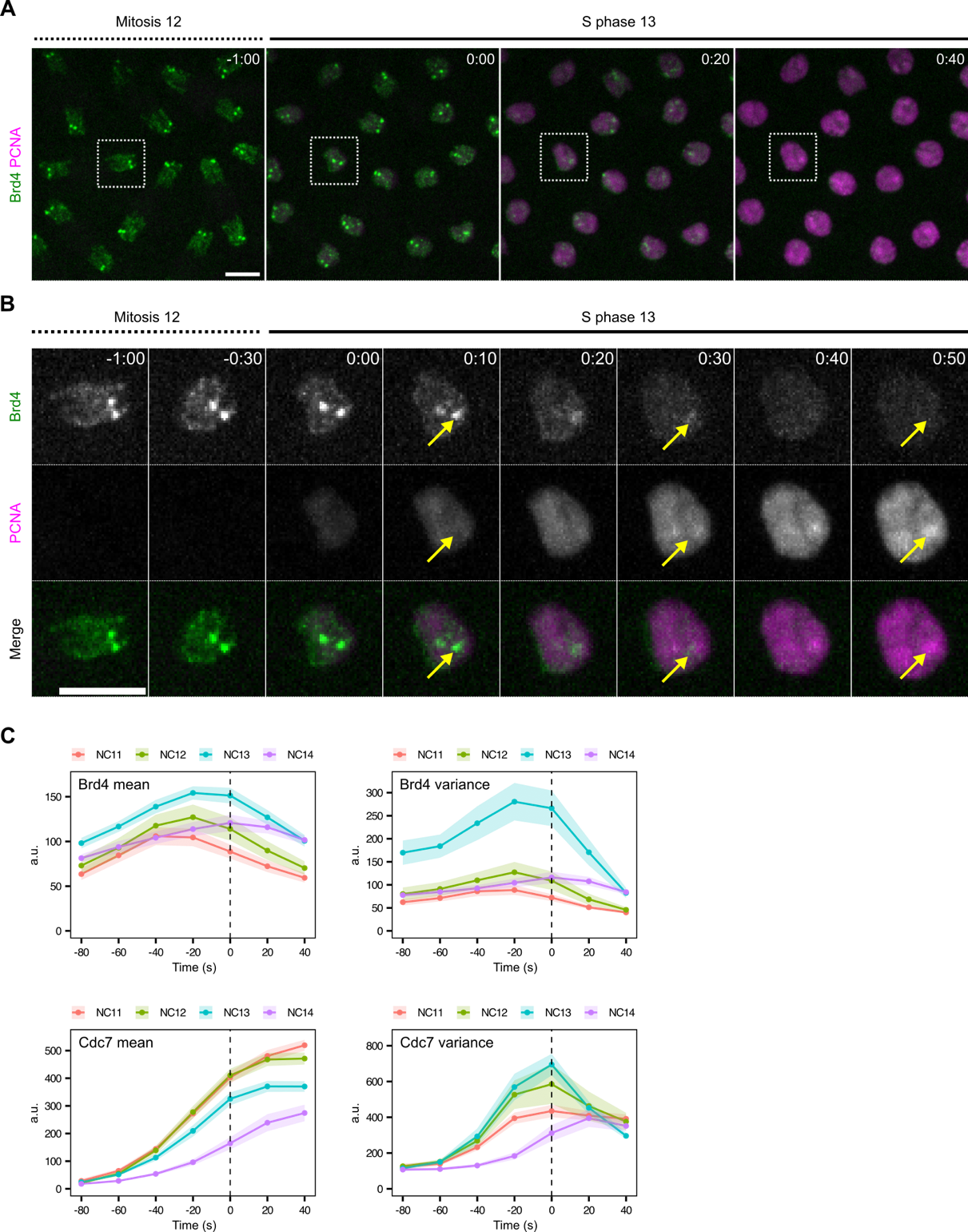
**

**Supplementary Figure 1. Additional data related to Figure 1. A, B** Representative stills from live imaging of endogenously tagged sfGFP-Brd4 and transgenic mCherry-PCNA in embryos transitioning from mitosis 12 into S phase 13. The boxes in panel **A** outline the same nucleus shown in panel **B**. Scale bars, 5 μm **C** Mean or variance of intensities for fluorescently tagged Brd4 or Cdc7 that are chromatin-associated during mitosis or nuclear-localized in S phase. Shaded areas represent SEM, n = 6 embryos. Time relative to the start of S phase is indicated. a.u., arbitrary unit.


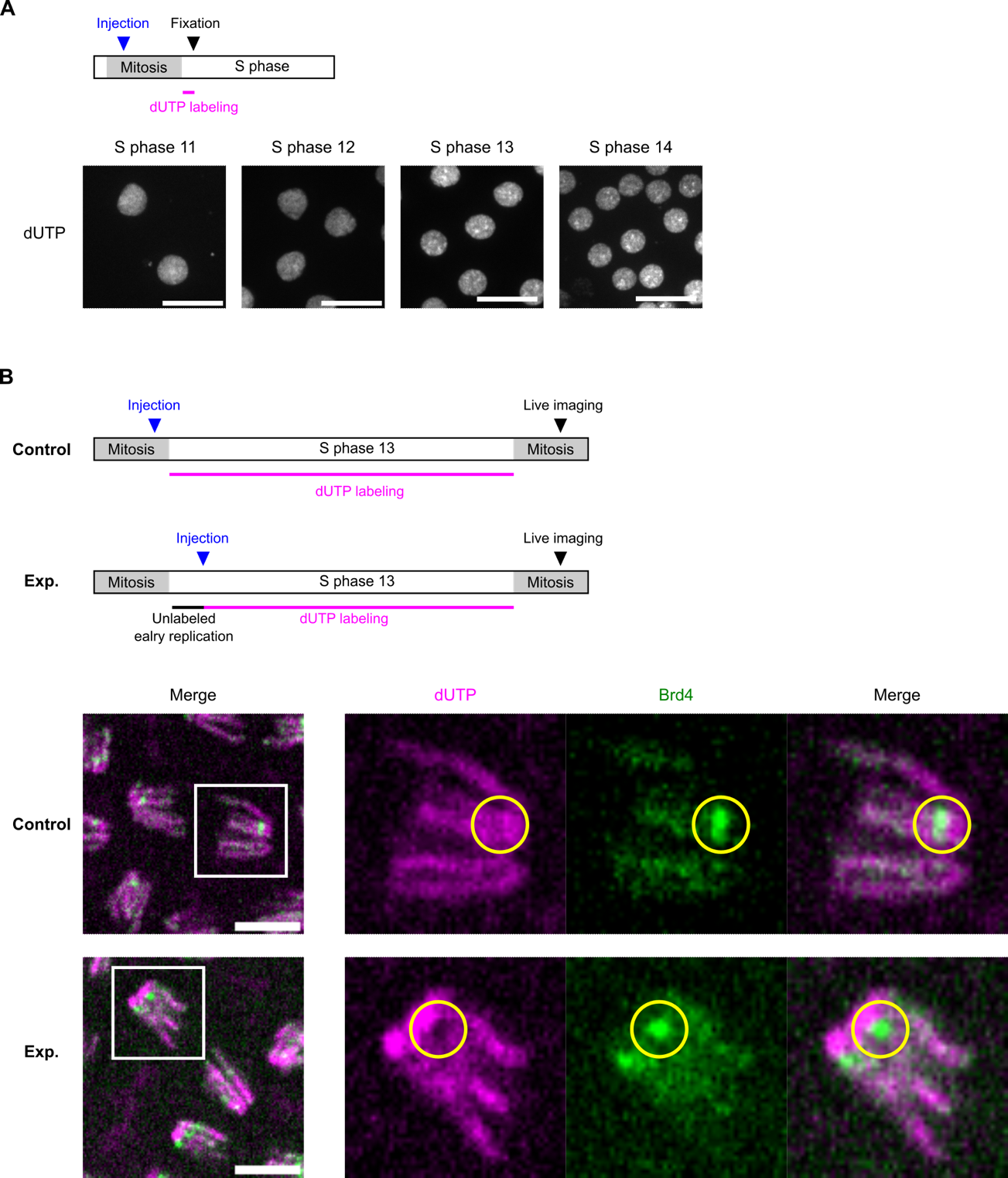


**Supplementary Figure 2. Additional data related to Figure 1. A** Representative images of fixed embryos subjected to short pulse-labeling with Cy5-dUTP at the beginning of S phases. Maximal projections are shown. Prominent early-replicating domains emerge after S phase 13. **B** Snapshots from live imaging of embryos injected with Cy5-dUTP either prior to the onset of S phase 13 (control) or ~3 minutes after S phase entry (experimental). In the latter embryo, Brd4-marked histone loci (yellow circles) did not show Cy5-dUTP signal, indicating that replication at these sites was completed prior to the injection window. Single-plane images are shown, which do not capture HLBs outside the focal plane. All scale bars, 5 μm.


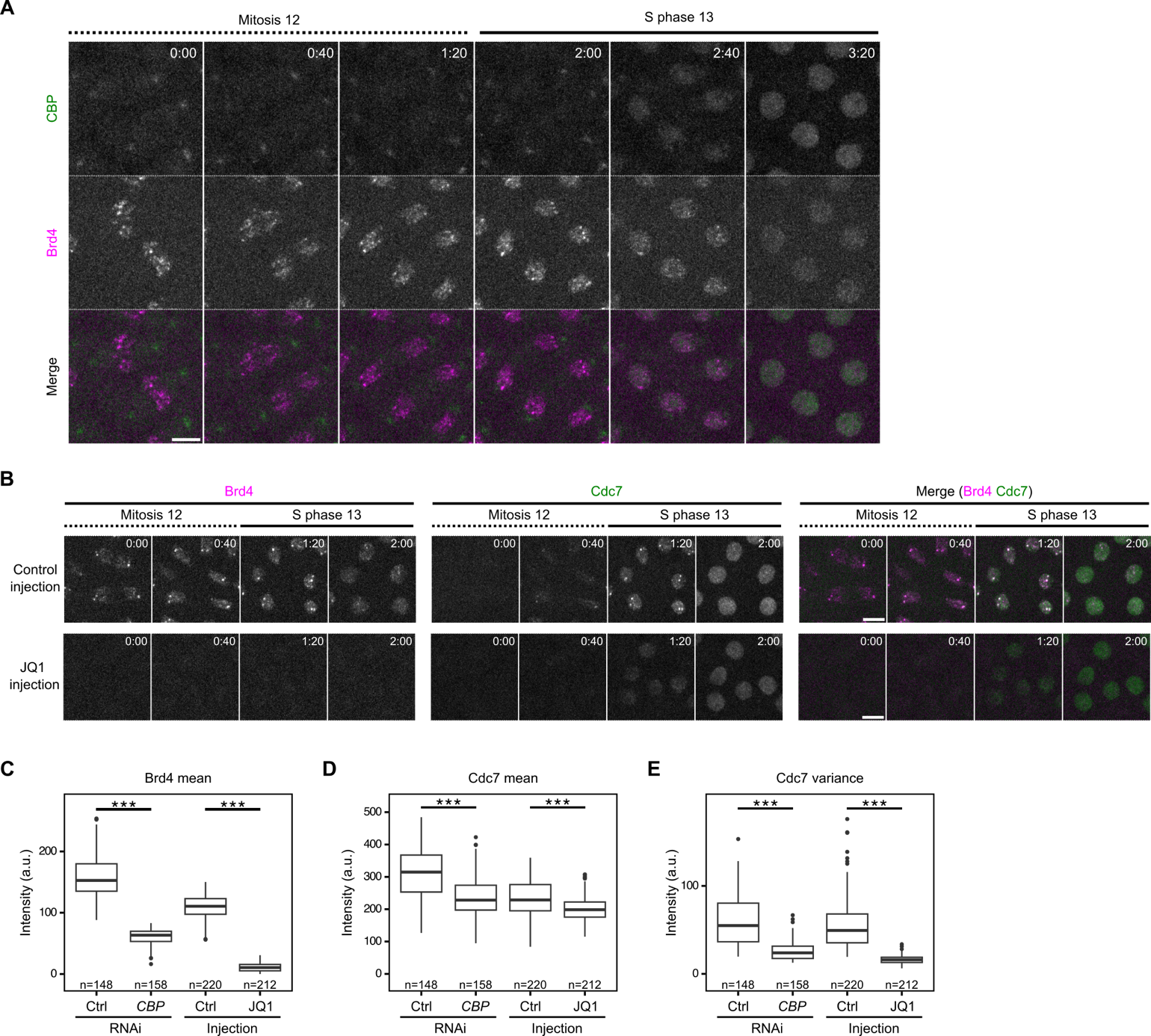


**Supplementary Figure 3. Additional data related to Figure 2. A** Representative stills from live imaging of mNeonGreen-CBP and mKate2-Brd4 during the transition from mitosis 12 into S phase 13. **B** Representative stills from live imaging of endogenously tagged mKate2-Brd4 and Cdc7-EGFP in embryos injected with 1% DMSO (control) or the Brd4 inhibitor JQ1. All images are maximal projections. Time relative to the start of each movie is indicated in minute:second. All scale bars, 5 μm. Images of JQ1-injected embryos shown here are identical to those in Figure 2C but are presented again for comparison with different control conditions. **C-E** Box plots showing the mean or variance of intensities for fluorescently tagged Brd4 or Cdc7 in nuclei at the start of S phase 13 in the indicated experiments. The central lines of the box plots represent median. Whiskers extend to 1.5 times interquartile range from the box. The number of nuclei (*n*) pooled from 5 embryos were indicated. ***, *P* < 0.001 by a two-sided Mann-Whitney U test with Bonferroni correction for multiple comparisons. a.u., arbitrary unit.


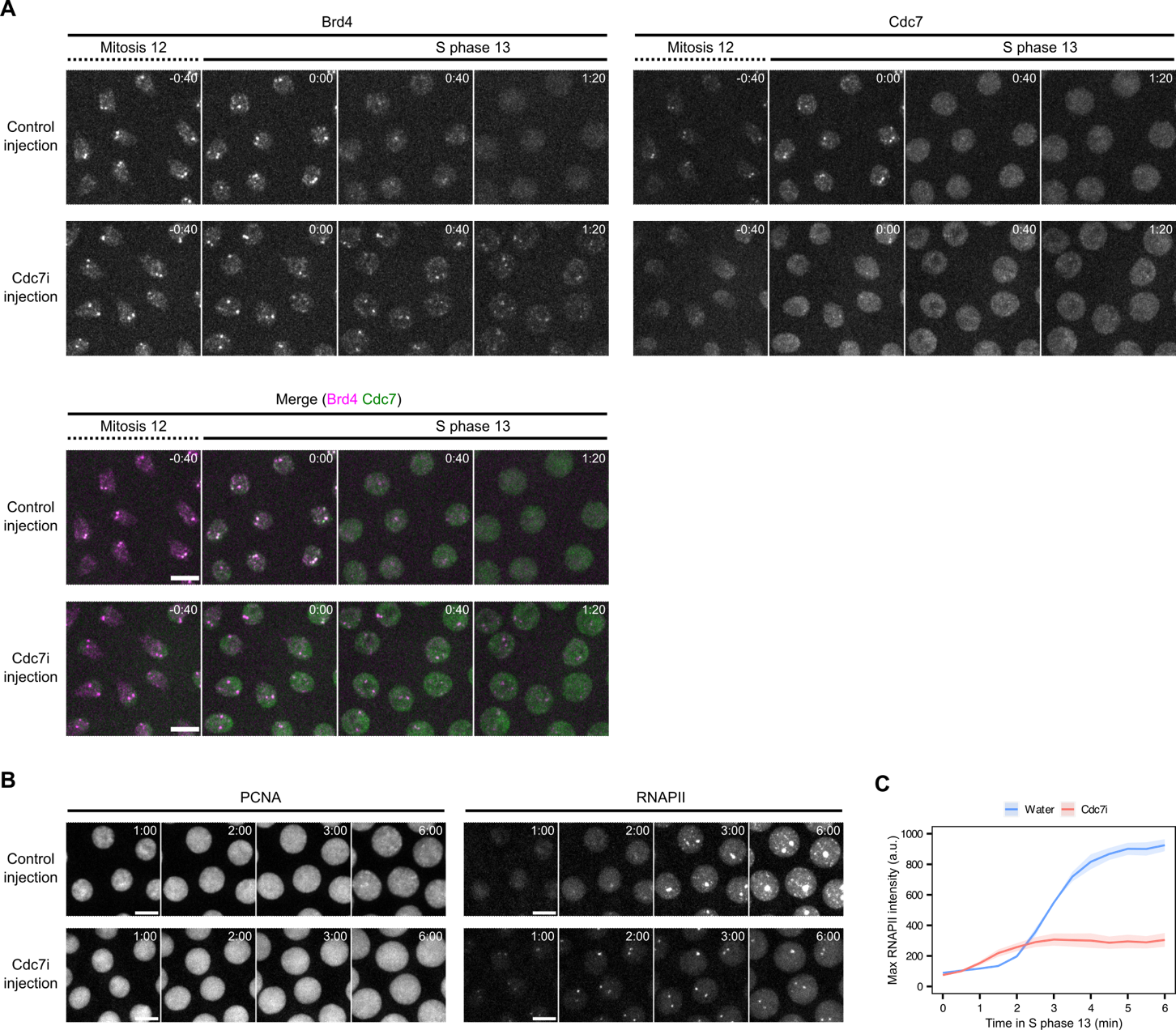


**Supplementary Figure 4. Additional data related to Figure 3. A** Representative stills from live imaging of endogenously tagged mKate2-Brd4 and Cdc7-EGFP in embryos injected with water (control) or Cdc7 inhibitor (Cdc7i) and transitioning from mitosis 12 into S phase 13. Time relative to the start of the movie is indicated in minute:second. **B** Representative stills from live imaging of mCherry-PCNA and EGFP-Rpb3 (a subunit of RNAPII) in control or Cdc7i-injected embryos during S phase 13. Recombinant mCherry-PCNA was co-injected with control or Cdc7i. Time relative to the start of S phase is indicated in minute:second. All images are maximal projections. All scale bars, 5 μm. Images for both control and Cdc7i-injected embryos are identical to those shown in Figure 3B; additional timepoints, larger fields of view, and the merged channel are presented here. **C** Quantification of nuclear EGFP-Rpb3 maximal intensity in control or Cdc7i-injected embryos during early S phase 13. The maximal intensity was measured in all nuclei and averaged per embryo. Shaded areas represent SEM, n = 3 embryos. a.u., arbitrary unit.


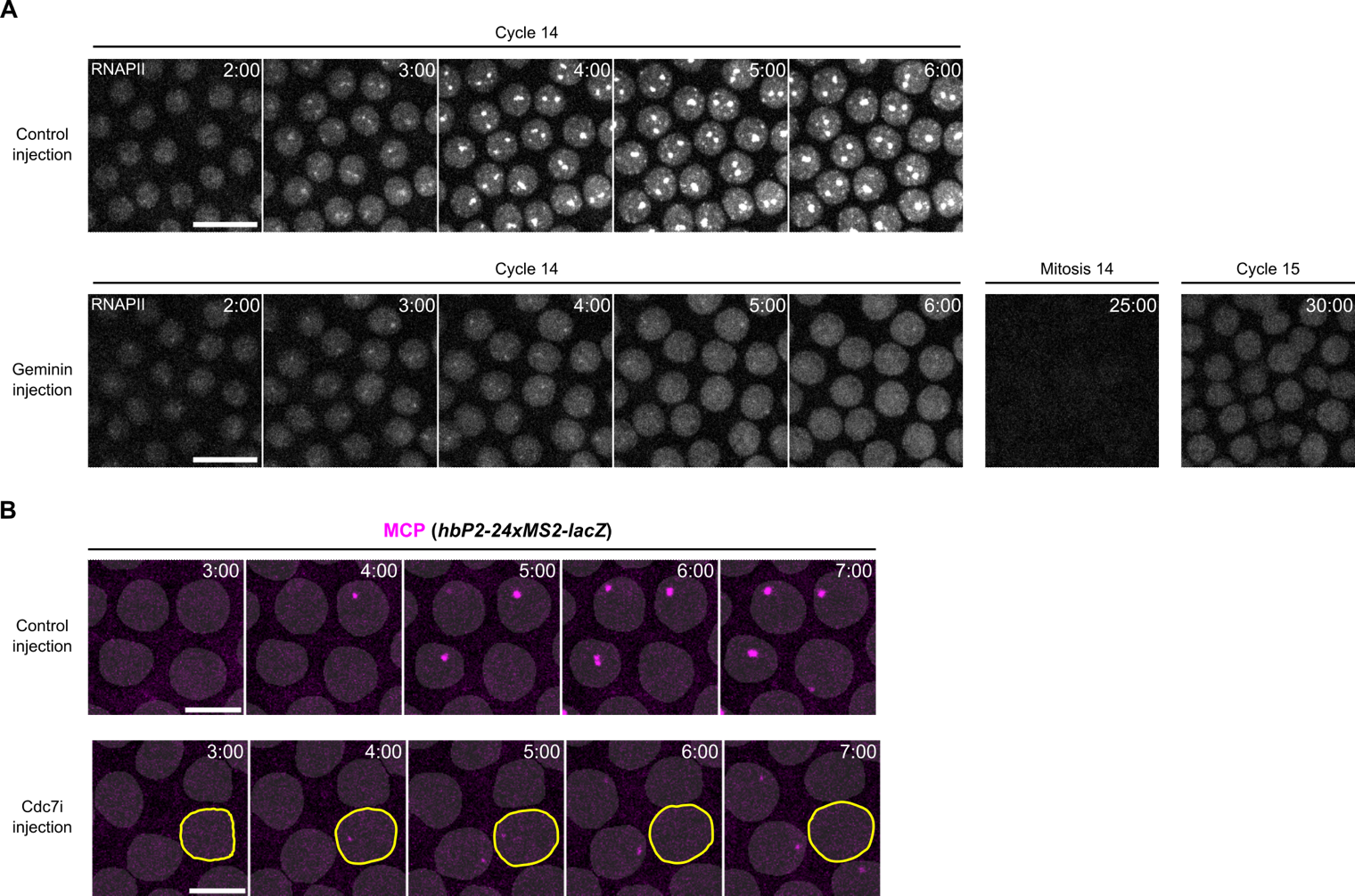


**Supplementary Figure 5. Additional data related to Figure 4. A** Representative stills from live imaging of mCherry-Rpb1 (RNAPII) in control or Geminin-injected embryos in S phase 14. The injection was performed in S phase 13. The Geminin-injected embryo underwent an extra cycle of synchronous division as a result of S-phase ablation. Time relative to the start of S phase 14 is indicated in minute:second. Scale bars, 10 μm. **B** Representative stills from live imaging of MCP-mCherry in control or Cdc7i-injected embryos with a *hbP2-24xMS2-lacZ* reporter. The binary masks of nuclei were generated from EGFP-Rpb3 images and overlaid on the MCP-mCherry images. Yellow circles highlight a nucleus whose MCP foci emerged and then disappeared within 3 minutes. Time relative to the start of S phase 14 is indicated in minute:second. Scale bars, 5 μm.


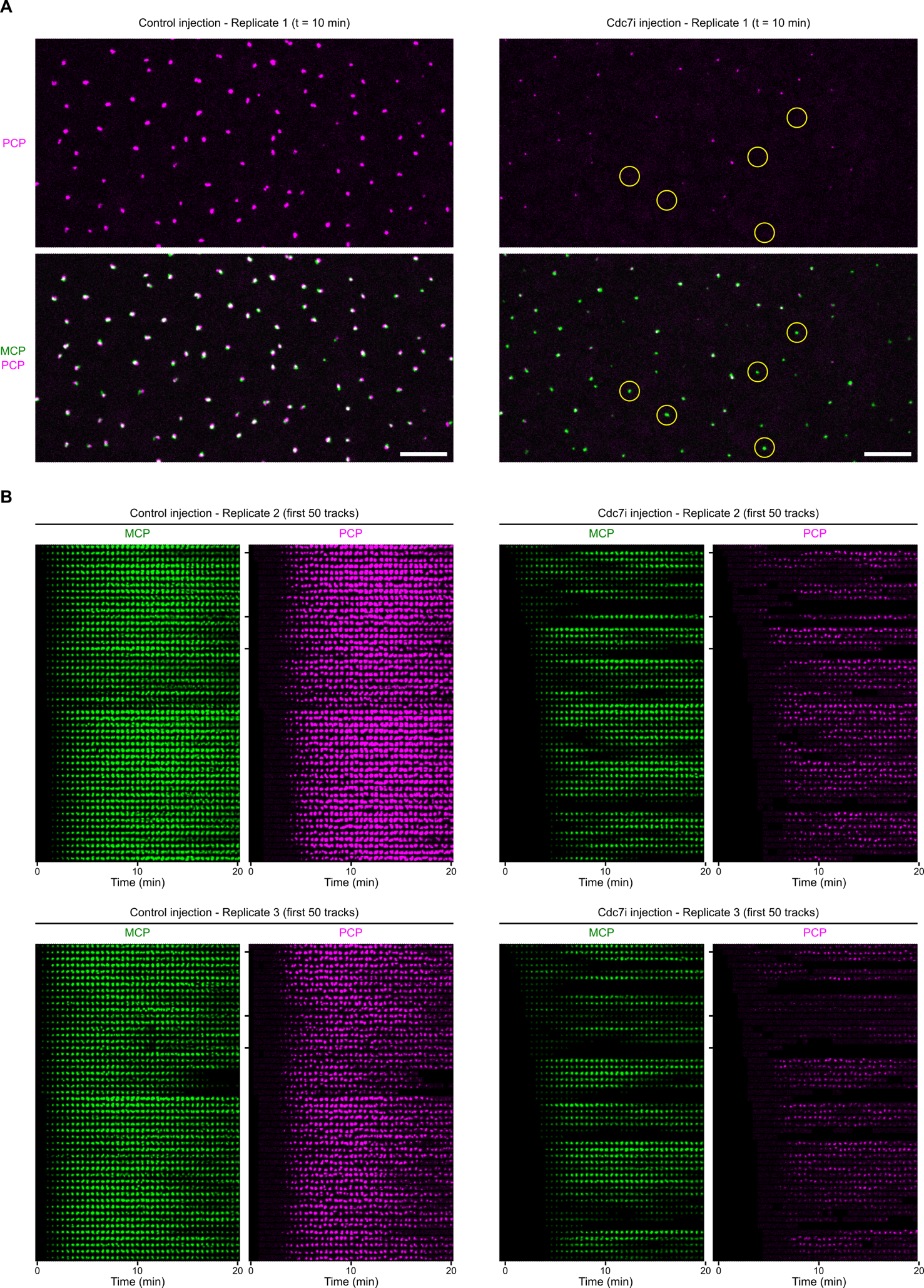


**Supplementary Figure 6. Additional data related to Figure 5. A** Snapshots from live imaging of MCP-GFP and mCherry-PCP in control or Cdc7i-injected embryos with the *hbP2-24xMS2-lacZ-24xPP7* reporter. Frames at 10 minutes after the initial apperance of MCP foci in the same embryo are shown. Yellow circles highlight the MCP foci that lack associated PCP foci. The embryos shown here are the same biological replicates as those in Figure 5B. Scale bars, 10 um. **B** Montages displaying signals from the transcriptional reporter in additional biological replicates of control or Cdc7i-injected embryos during S phase 14. The top 50 spots with the earliest MCP appearance are displayed.

| **Supplementary Table 1. *Drosophila melanogaster* lines used in this study** | |
| --- | --- |
| **Genotype** | **Source** |
| *w, sfGFP-Brd4, mCherry-Rpb1* | Cho and O'Farrell, 2023 |
| *w, mKate2-Brd4* | This paper |
| *w, Cdc7-EGFP* | Seller and O'Farrell, 2018 |
| *w, mKate2-Brd4, Cdc7-EGFP* | This paper |
| *w, mNeonGreen-CBP* | This paper |
| *w, mKate2-Brd4, Cdc7-EGFP;; UASp-shRNA.w* | This paper; Bloomington Drosophila Stock Center (#35573) |
| *w, mKate2-Brd4, Cdc7-EGFP;; UASp-shRNA.nej* | This paper; Bloomington Drosophila Stock Center (#36682) |
| *w, mKate2-Brd4, Cdc7-EGFP;; Mat-tub-Gal4* | This paper; Bloomington Drosophila Stock Center (#7063) |
| *w; EGFP-Rpb3; nos-MCP-mCherry* | Cho et al., 2022 |
| *yw; hbP2-MS2-lacZ* | Bloomington Drosophila Stock Center (#60338) |
| *w; nos-NLS-mCherry-PCP, His2Av-eBFP2/CyO; MCP-GFP* | Fukaya et al., 2017 |
| *hbP2-MS2-lacZ-PP7* | Fukaya et al., 2017 |
